# Motivations for Relationships as Sources of Meaning: Ghanaian and South African Experiences

**DOI:** 10.3389/fpsyg.2020.02019

**Published:** 2020-08-21

**Authors:** Marié P. Wissing, Angelina Wilson Fadiji, Lusilda Schutte, Shingairai Chigeza, Willem D. Schutte, Q. Michael Temane

**Affiliations:** ^1^Africa Unit for Transdisciplinary Health Research, Faculty of Health Sciences, North-West University, Potchefstroom, South Africa; ^2^Inclusive Economic Development Unit, Human Sciences Research Council, Cape Town, South Africa; ^3^Department of Psychology, Faculty of Humanities, University of Pretoria, Pretoria, South Africa; ^4^Centre for Business Mathematics and Informatics (BMI), Faculty of Natural Sciences, North-West University, Potchefstroom, South Africa

**Keywords:** African perspectives, meaning, motivations, relationships, interconnectedness

## Abstract

Afrocentric paradigms reflect assumptions of the overarching importance of interconnectedness and social bonds in meaningful experiences. It is, however, not known if types of relatedness vary in importance as meaning sources in the subjective experiences of laypeople, or what the reasons are that they ascribe to the importance of relationships. The empirical and theoretical substantiation of philosophical assumptions is needed to provide a scientific basis for appropriate well-being interventions in African contexts. Therefore, this study aimed to empirically explore the relative importance of various types of relationships as sources of meaning and in particular *why* relationships are important to laypeople in relatively collectivist African contexts. Using a bottom-up qualitative approach with quantification of responses, this study explored how prominently relationships featured as meaning sources compared to other domains of life and then, in particular, the motivations for the importance of various types of relationships as found in four African samples: a Ghanaian urban group (*n* = 389), a South African multicultural, English-speaking urban group (*n* = 585), and two South African Setswana-speaking groups (*n* = 512 rural, *n* = 380 urban). Findings showed that the relational domains of life, namely, family, interpersonal relations, spirituality/religion, and community/society, made up a large proportion of responses on what provides meaning in life−in particular family and spirituality/religion with community/society occurring the least. The reasons for meaning experienced in various relationship types included domain-typical relational descriptors, such as contributions made or rewards received. However, many intrapersonal motives also emerged: inner well-being, happiness, joy, a sense of competence, and own growth. Material needs and harmony also surfaced as motivations for relational importance. Findings are aligned with African philosophical perspectives as far as the importance of relationships and the value attached to spirituality/religion are concerned, but contributed additionally by showing that different types of relationships vary in importance: close relationships are more important than community/societal relationships. Unearthing the reasons for the importance of relationships points toward a dialectic pattern of African individualism–collectivism in which independent and interdependent orientations flow together. Such knowledge is vital for the promotion of mental health and well-being in these contexts.

## Introduction

Meaning in life is about what people value most. Various sources of meaning in life had been identified, with relationships (e.g., with family, close others, community members, divinity, and ancestors) being one of the most often cited meaningful things for people across cultures (e.g., Lambert et al., 2013; Onyedinma and Kanayo, 2013; Wissing et al., 2014; Delle Fave et al., 2016; White, 2017; Appiah-Sekyere, 2018; Wissing et al., 2019b). However, the importance and significance of specific relationships can vary based on the cultural context and life situatedness (Khumalo et al., 2012; McCubbin et al., 2013; Onyedinma and Kanayo, 2013; Thomas et al., 2017).

Delving deeper into *why* relationships or particular kinds of relationships are meaningful to people (versus only *that* they are important for meaning, well-being, and other positive outcomes) had not been explored in an African context as far as we can ascertain. The *why* in this sense is seeking the reasons or motivations for the indications of *what* is important for meaning. Although it is well-known that horizontal (interpersonal) as well as vertical (spiritual) relationships are very important in African collectivist contexts, given specific cultural values (Graham, 1991; Mbiti, 1991; Mkhize, 1998; Baloyi and Mokobe-Rabothata, 2014; Nwoye, 2017; Cram, 2018), very little is still understood about what laypeople exactly experience and describe as the reasons/motivations for the importance of these relationships in meaning–construction/detection in their specific contextual situatedness. For purposes of this study, we focus from a bottom-up perspective on the reasons why relationships are meaningful to laypeople from Ghana and South Africa as subjectively experienced. Underlying the need for this study is the scarcity of reliable and contextually relevant well-being theories and interventions in these contexts, developed from understanding individual lived experiences as opposed to indiscriminate transference of individualist Western theories and practical intervention tools or simply accepting all-out collectivistic assumptions. Apart from building on other studies, our work also adds new knowledge with regard to the relative importance of various types of relationships in the African context, as well as the grounding of subjective notions on meaning and relationships in these specific contexts. Thereby we can provide empirical backing for, and testing of, traditional philosophical assumptions and existing theoretical perspectives emerging from Africa. To avoid the process of “othering” (Makhubela, 2016, p. 1), both African and Western perspectives will be taken into account where relevant.

### Meaning

Meaning is conceptualized in many cultural contexts as being informed by values and as being associated with (especially relational) well-being outcomes. This is the case from African as well as Western philosophical perspectives, but also in some theories and empirical studies (Ngubane, 1977; Slife and Richardson, 2008; Nwoye, 2015; Fowers, 2017). Metz (2011) proposed a moral theory of human rights and dignity. He builds this model on the interpretation (meaning) of the indigenous concept of “Ubuntu” (a person is a person through other persons). He contended that “typical human beings have a dignity by virtue of their capacity for community, understood as the combination of identifying with others and exhibiting solidarity with them” (Metz, 2011, p. 532; cf. also Metz, 2013). Thus, the African value of Ubuntu is interpreted/given meaning as reflecting a specific valued quality of relatedness. Western research had empirically explored the structure of meaning, the antecedents or sources of meaning, and the reasons or motivations for sources of meaning (dynamics). The present study focuses on various relationships as sources of meaning, the reasons for relationships as a source of meaning in an African context, and how these may fit into more indigenous models of well-being.

A leading Western model of meaning is that of Steger et al. (2009), Steger (2012). Comprehension and purpose are seen as the main *structural components* of meaning. More recently, Martela and Steger (2016) suggested that coherence, purpose, and significance (as affectively evaluated) are the three main facets of meaning. These facets resemble those proposed by George and Park (2016), described as cognitive, motivational, and affective/existential mattering. Models of meaning thus typically refer to comprehension/sense of coherence (cognitive facet) and/or purpose (motivational function), as well as an affective–experiential (subjective-evaluative facet) as main components of meaning, but “significance” and “mattering” are also at times associated with spiritual denotations.

Various specific perspectives on the *sources of meaning* (often empirically supported) can be found, and in many of these studies, relationships are seen as an important source (e.g., Leontiev, 2007; Lambert et al., 2013; Ryff, 2014). All of these perspectives refer, in one or the other way, to intrapersonal, interpersonal, and/or transpersonal relational aspects. Based on bottom-up qualitative studies conducted across several countries, Delle Fave et al. (2013) categorized meaning attributions to life domains as espoused in previous quality-of-life studies (Whoqol Group., 2004) and developed a coding system for qualitative data in this regard. The main identified life domains in which meaning was found and included in this coding system are family, work, standard of living, interpersonal relationships, health, personal life, leisure, spirituality/religion, society issues, community issues, and life in general. Several of these are relational, namely, family, interpersonal relationships, society, community, and spirituality. Prominent sources of meaning emerging from Mason’s (2013) study among university students in South Africa included religion, relationships, and education. Underlying these sources is the sense of connectedness, direction, and the need to give back as well as succeed.

Although there are common motives for meaning in life across Western and African contexts, there are some less emphasized differences worth pointing out. For example, in a study among a South African sample, Mason (2015) noted that the belief in a bright future emerging from using innate resources and talents, as well as outstanding academic achievement, was related to making meaning out of life. In contrast to Western findings on meaning in life, Mason (2015) found that emphasis on material needs and goals was linked to meaning in life in the South African context. Furthermore, according to Mason (2013), African metaphors do manifest in meaning attributions. Noteworthy are those referring to Ubuntu and Batho Pele (meaning “people first”), which point to connectedness and self-effacing while putting others first (Senghor, 1970; Mason, 2013). Meaning could subsequently be described as an integration of individual responsibility, a collective social connectedness, as well as concern for others’ well-being (Mason, 2013).

In a study by Nell (2014), relationships, especially with family, as well as hope, education, achievement, and religion, were found to be sources of meaning among a sample of university students in South Africa. Moreover, the qualitative analysis revealed that most of these sources were valued as a means to an end rather than for their intrinsic qualities. The sources of meaning did not lie in a present career or set of professional achievements as much as in anticipation of the eventualities and the benefits thereof (Nell, 2014). This finding seems to contradict Mason (2013, 2015), who indicated that underlying the attributions of meaning in the African context is a sense of connectedness, direction, relational dependency, and the need to give back. Relatedly, Fallon (1999), De Witte (2003) noted that wealth in the Akan culture in Ghana is more or less a social glue that has the potential of forging kin relations where there are no blood ties. This implies that the emphasis on material things and socioeconomic factors in the experience of meaning might be linked to attributions of maintaining or forging kin relations, rather than preoccupation with need satisfaction.

Even when other domains of life feature in people’s experiences and sources of meaning in African contexts, relational motives are often implicit. For example, further work on meaning in life has been explored in Ghana as understanding perceptions of the “good life.” Dzokoto et al. (2019) explored perceptions of meaning and happiness in a Ghanaian context and noted that work-related meaning was associated with doing significant work that facilitates personal growth and contributes to the greater good. The greater good was interpreted as being able to provide for one’s family. Among working women in South Africa, meaning in life was derived not only from intrinsic satisfaction and subjective experiences of power and authority that their work provided, but also from work-derived identity, experiences of self-worth, and impact on others through service and social connections afforded by their work (Person et al., 2016). The experience of meaning in life from work that impacts others is consistent with interdependent cultural contexts, where practices and beliefs that build and maintain connections with others and cordiality are encouraged.

### Relationships

Relationships and interconnectedness play an important role in African worldviews (Onyedinma and Kanayo, 2013; Owusu-Ansah and Mji, 2013) and in interconnectedness metatheoretical perspectives in the West (e.g., Stroink and DeCicco, 2011; Helne and Hirvilammi, 2015). Owusu-Ansah and Mji (2013) contended that a person pursues individual and collective harmony to become a whole person. This idea resonates with findings of Delle Fave et al. (2016), who established in a multicultural cross-country study that the psychological content most often mentioned as meaningful referred to harmony and balance. Also, Durie (2004) found that health was basically about relationships in the case of rural South African women and that health requires harmony with others. Harmony thus reflects a positive or ideal quality of relatedness. From a Western perspective, the centrality of relationships in human life for the experience of meaningfulness had also been argued by Ryff and Singer (1998, 2000) and Ryff et al. (2004) showing the health and well-being benefits from loving relationships and purposeful living. Relationships are furthermore linked to a variety of other indicators of well-being (cf. Ogbonnaya, 1994; Selvam, 2013; Nwoye, 2017; Harrell, 2018; Warren and Donaldson, 2018).

Positive relationships and emotions are part of being well as viewed in African and Western perspectives, but the specific reasons why relationships are important for meaning in life may differ within the parameters of opportunities and boundaries of the specific context (Greenaway et al., 2018; Wilson et al., 2019a). The expression of these relationships had been shown to differ between Western individualist and Asian collectivist contexts (Markus and Kitayama, 1991). However, Hermans and Kempen (1998) cautioned that there is a need to interrogate traditional dichotomies that separate the West from the rest of the world implying internal homogeneity and external distinctiveness. Following this line of argumentation and paucity of research in Africa, we cannot assume that African collectivist orientations are the same as Asian collectivist perspectives and must guard against assumptions of internal homogeneity (Wissing and Temane, 2013). This necessitates empirical investigation into reasons for the importance of relationships in context. Cultural differences in value systems and norms may affect the importance individuals attribute to specific daily activities and social roles (Triandis, 1994; Berry, 1997) and thus relational types.

Samuel et al. (2014) proposed that relationships from an African perspective are defined in interconnectedness, interdependence, sense of solidarity, and belongingness. Social connectedness lies at the heart of care and support of African people (Stavrova and Luhmann, 2016). Thus, the relational nature of African cultures includes “wholeness, community, and harmony, which are deeply embedded in cultural values” (Owusu-Ansah and Mji, 2013, p. 2). To be human is to be in the community, participating in beliefs, ceremonies, rituals, and festivals that give a sense of belonging (Mbiti, 1991). A community is emphasized more than the member’s individuality, and this creates a chain of interrelationships. These assumptions can create the expectation that communal types of relatedness may be more important than close personal relationships, which is of primary importance in the West, but this had not been empirically explored as yet. More so, these communal types of relationships might be further complicated with obligatory reciprocal interactions in the African culture. The autonomy and rights of the person are enjoyed in a communal relationship (Nussbaum, 2003; Nwoye, 2004). A person is defined through community and in relationship with other people.

The cultural belief system and role obligations also uphold the connections, relationships, and the purpose and meaning in life for African people (Pflug, 2009; Wissing et al., 2014). Ratele et al. (2004) supported the notion that there is a collective sense of responsibility among African people and that community members are bound together by a reciprocal understanding of their roles and responsibilities. Nwoye (2004), Zwane et al. (2012), Onyedinma and Kanayo (2013) were also of the opinion that relatedness is seen in engaging in daily activities that foster respect and cultural practices of the particular culture of African people. For example, cultural practices of raising children according to cultural values are core to sustaining the bonds and relationships among African people (Owusu-Ansah and Mji, 2013). It is the responsibility of the community to educate children in an African tradition. Mbiti (1991, p. 75) also mentioned that “It takes the whole village to raise a child.” This sense of solidarity and community wholeness enables African people to build strong, meaningful relationships (Onwubiko, 1991; Uzukwu, 1996). We should, however, also recognize that individuals and communities may seamlessly change over time and that the manifestations of meaning and relationships may change over time, for example, through urbanization.

Relationships of African indigenous people are also described in various facets and contexts besides familial and community connectedness and typically include spiritual and religious facets, as well as a connectedness to nature (cf., Onyedinma and Kanayo, 2013; Nwoye, 2017). Cram (2018) viewed the “relationships world” of African indigenous people as that which exists between people, people and their environment, and people and their spirituality. In this regard, people are intimately connected to nature and spiritual dimensions, which form part of their identity. Thus, spirituality is regarded as the thread that connects humans to one another, nature, and to the Creator. These relations provide individuals with a sense of purpose (Nwoye, 2017). Onyedinma and Kanayo (2013) defined African human relationships as consisting of two dimensions, horizontal (with fellow humans) and the other vertical (with the divine). Nwoye (2017) also believed that, in African culture, there is a link between the self, the community, and the spiritual world, including ancestors, with spirituality being deeply embedded in the development of relationships and a healthy life cycle of Africans (Wheeler et al., 2002; Greeff and Loubser, 2008). Ohajunwa and Mji (2018) highlighted that spirituality is rooted in the connectedness to each other and that well-being is acquired through a harmonious connection with God, other people, and nature. Given previous assumptions, and some empirical findings of the importance of spirituality in the African context, it can be expected that laypeople value vertical relatedness more than what was previously found in Western studies (e.g., Delle Fave et al., 2011).

### Relationships in Meaning-Making Processes

One of the prominent sources of meaning is relationships as found in several studies. Lambert et al. (2013) argued that social relationships represent an important conduit for the expression and experience of meaning. Among other sources of meaning in life, relationships form the center of the present study because not only do relationships exist in every culture and context, but they also form a crucial part of the African worldview (Ngubane, 1977; Nwoye, 2015). Despite the central role of relationships, few studies have explored its role and the mechanism through which it promotes meaning in life. Wilson et al. (2019a) suggested that, among a sample of South Africans, family ties were linked to meaning attributions because they shape an individual’s disposition, orientation, and personality. Moreover, other forms of vertical and horizontal connectedness were linked to identity formation. In essence, our relationships reflect both who we are and who we want to be. In understanding goal motivations in rural South African communities, Wilson et al. (2019b) noted those goal motivations (which are key components of meaning-making) had a robust interdependent focus embedded in existing relational ties. Themes emerging from the study, including financial security, social needs, generative caring, and relational goals, all pointed to how relationships function in providing a coherent and systematic view toward the world. In line with these findings, Wilson et al. (2019a) found that relational well-being among the aged in both rural and urban South African samples highlighted personal identity as a function of relationships.

Apart from philosophical perspectives on the importance of relationships in African meaning systems and some descriptive empirical findings, very few theoretical models have been proposed in this regard. Relationships have been mostly empirically explored on the interpersonal level, but it can also refer to intrapersonal, social, transpersonal, and physical contextual levels as in the meaning and relational well-being (M&RW) model by Wissing et al. (2019b). Another critical well-being scholarship conducted in the global south expands relationships to include the broader sociocultural context in which people aspire to be well. Such an expanded focus allows the investigation of “underlying social, governmental, political, and cultural structures and processes” (White, 2015, p. 11).

Mahali et al. (2018) argued for a conceptualization of well-being as existing in relationships as a perspective for understanding well-being in the African context. White (2010) promoted the idea of “relationship […] being at the center of well-being analysis and politics” (p. 15) within the global south. In their article on dynamics of social support, Wilson and Somhlaba (2016) found that among adolescents in the Northern region of Ghana, relationships with extended family members and peers engendered eudaimonic well-being through fostering self-belief in the adolescent’s ability to succeed. Salifu Yendork and Somhlaba (2017) also indicated that, among orphanage children in Ghana, support received from peers increased emotional well-being. Existing social ties were identified as enabling orphans to make positive meaning out of orphanage placement, promote their well-being, and facilitate adjustment to orphanhood (Salifu Yendork and Somhlaba, 2017). Such empirical findings can be building blocks for integrative conceptual models of relational well-being and meaning. Hermans and Kempen (1998) also indicated the need to view psychological experiences through the lens of emerging hybridization, heterogeneous global systems, and increasing cultural complexity. As a result, there is a need for further research to understand the reasons why relationships are so important in experiences of meaning in life.

### The Present Study

The importance of relationships in African values and meaning systems is abundantly described in philosophical perspectives, but very little empirical studies in well-being research had been conducted, and there is a gap in knowledge about the relative importance of different types of relatedness and the *reasons* for the importance of relationships. Despite an abundance of philosophical assumptions, theorizing about the dynamics of relatedness had been neglected in the African context. Developing African cultural models in psychology is hinged on validating philosophical assumptions on the importance of relationships, with extensive empirical research on why relationships are important in the first place. Therefore, this study empirically tapped into the verbal responses of laypeople elicited by two semistructured questions on what the most meaningful things are for them and why they find these things meaningful. Then, we applied a developed coding process to code each verbal response after which coded answers were transformed into quantitative data. We will focus from a bottom-up perspective specifically on the reasons why relationships are meaningful for four groups of laypeople from Ghana and South Africa. The two samples were selected because they represent two distinct but related settings: one a developing middle-income country with a strong racially divided context (South Africa), and the other an upper low-income country with a higher level of cultural integration (Ghana). These two contexts have the potential of unearthing unique dynamics for enhancing our understanding of the relational motives for meaning.

Our specific aims are to: (1) determine the relative importance of various relational domains (types of relationships) and (2) gain a deeper understanding of *why* relationships, in particular, in the relational domains of family, interpersonal relations (close, but not family, e.g., friends, neighbors), spirituality/religion, and community/society, are meaningful to people. As this is an exploratory descriptive study, no specific hypotheses are stated. The method used for all four samples is described below.

## Materials and Methods

### Samples and Design

Four samples were selected. Samples 1 and 2 were adults from urban areas in Ghana and South Africa, respectively, in which participants had at least a secondary school education and were 18 years or older. Participants completed the questionnaires in English. The South African sample was multicultural. Samples 3 and 4 were Setswana-speaking older adults with lower educational levels from rural and urban areas in South Africa, respectively. They were selected in line with inclusion criteria for the Prospective Urban and Rural Epidemiological Study for South Africa (PURE-SA) (cf. Teo et al., 2009). Participants in samples 3 and 4 completed the questionnaires in an interview format administered by bilingual interviewers. Interviews were conducted in the indigenous language spoken in the area, Setswana. Ghana is located in a subregion of West Africa and is situated on the Gulf of Guinea and the Atlantic Ocean. South Africa is at the southernmost point of Africa and part of the sub-Saharan area. While both Ghana and South Africa are relatively well-developed countries, they represent different regions of the African continent and allow for diversity, which motivates the selection of these sites. The authors were also knowledgeable about the context and languages spoken in these areas having previously been involved in other larger projects in these areas.

In each of the four samples, data were collected in a one-off cross-sectional design including the same semistructured, open-ended questions to which written responses (samples 1 and 2) or fieldworkers capturing verbal responses (samples 3 and 4) were given by participants. Data gathering for samples 2, 3, and 4 formed part of the FORT3 research program (Wissing, 2008/2012). In the case of samples 3 and 4, FORT 3 data were gathered in an overlap with the 10-year follow-up of the longitudinal PURE-SA study (Teo et al., 2009) and were considered as cross-sectional for the purposes of this study. The semistructured questions used in English in samples 1 and 2 were translated to Setswana for samples 3 and 4 using a forward and back-translation method involving bilingual (English and Setswana) academics and were checked by lay Setswana mother tongue speakers for clarity and comprehension.

### Participants

See Table 1 for a description of the participants per sample.

**TABLE 1:** Demographic profile of the four samples.

Variable	Ghana (*n* = 389)	Multicultural SA (*n* = 585)	Setswana SA (rural) (*n* = 512)	Setswana SA (urban) (*n* = 380)
**Gender**				
Male (%)	54.88	38.01	28.91	68.95
Female (%)	45.12	61.99	71.09	31.05
No. missing	10	1	0	0
**Age**				
Mean (SD), range	41.42 (9.36), 30–62	42.15 (11.51), 18–84	58.10 (8.58), 42–87	60.23 (9.97), 43–103
No. missing	10	1	3	1
**Level of education**				
None (%)	0.00	0.00	27.07	15.55
Primary (%)	0.00	0.00	58.79	39.41
Secondary (%)	51.73	37.87	13.33	44.77
Tertiary (%)	41.07	30.29	0.81^a^	0.27^a^
Postgraduate (%)	7.20	31.84	−^a^	−^a^
No. missing	14	4	17	7
**Marital status**				
Single (%)	39.14	25.17	25.66	44.39
Married (%)	45.58	63.72	40.40	27.01
Cohabitating (%)	4.83	3.30	15.35	0.80
Separated/divorced (%)	4.56	5.90	4.04	5.08
Widowed (%)	5.63	1.91	14.55	22.73
No. missing	16	9	17	6
**Practicing religion/member of religious group^b^**				
No (%)	2.93	7.49	50.40	70.08
Occasionally (%)	23.20	26.66	−^b^	−^b^
Regularly/yes^b^ (%)	73.87	65.85	49.60	29.92
No. missing	14	11	10	9
**Employment status**				
Unemployed (%)	0.82	21.34	96.42	82.06
Employed (%)	99.18	78.66	3.58	17.64
No. missing	25	257	9	1

*SA, South Africa. ^a^ For the two Setswana SA samples, basic tertiary education and postgraduate tertiary education were not distinguished in the questionnaire. ^b^For the Ghana and multicultural SA groups, the question was whether the participant practice religion, with responses “no,” “occasionally,” or “regularly” allowed. For the Setswana SA samples, the question was whether the participant was a member of a religious group (e.g., church group, etc.), with responses “no” or “yes” allowed.*

### Data Gathering

#### Sociodemographic Questionnaire

For all four samples, the sociodemographic questionnaire included gender, age, educational level, marital status, practicing of religion, and employment status.

#### Semistructured, Open-Ended Questions

The present study used two open-ended questions on meaning from the Eudaimonic–Hedonic Happiness Investigation (EHHI) (Delle Fave et al., 2011), namely, “Please list the three things that you consider most meaningful in your present life” (meaning sources) and “For each of them, please specify why it is meaningful (try to be as specific as possible)” (reasons).

### Procedure

After obtaining ethical approval for the various studies, participants were selected according to the inclusion and exclusion criteria. Trained fieldworkers invited participants to answer the semistructured questions mentioned previously, as part of a larger battery of questionnaires (for samples 3 and 4, fieldworkers were fluent in English and Setswana). In samples 1 and 2, the participants provided written answers, whereas in the cases of samples 3 and 4 (with generally lower educational levels than those in samples 1 and 2), participants answered verbally, and their responses were captured in writing by the fieldworkers. All responses were then coded, categorized and clustered in life domains, and then transformed to quantitative data for further analyses (see details in “Data Analysis”).

### Ethical Considerations

Trained fieldworkers obtained written informed consent from participants (in cases where participants in samples 3 and 4 were illiterate, a thumbprint was used to confirm consent) and made sure that participants understood what was expected from them, that participation was voluntary, and that they had the right to withdraw at any point in time during data gathering. Anonymity was safeguarded by separating the signed consent forms from the corresponding answer sheets, and all confidentiality concerns were upheld. The University of Ghana Ethics Committee for Human Research approved of the study reported in sample 1 (approval no. ECH 086 16-17) and the Health Research Ethics Committee of the North-West University, South Africa, approved the studies reported in samples 2, 3, and 4 (approval nos. NWU 00002-07-A2 and NWU-00016-10-A1).

### Data Analysis

Participants’ written responses to the semistructured questions (qualitative data) were coded using the formalized manual for a coding system developed by Delle Fave et al. (2011). Coded data were then quantitatively analyzed. The EHHI coding system was developed by a research team based on responses to various semistructured questions on happiness, goals, and meaning as obtained from participants in eight countries and over time elaborated and refined with data collected in several more countries from six continents (Delle Fave et al., 2011, 2016). When developing the coding system, the process started with the reading and rereading of the text, extracting meaning units, formulating names or codes, the grouping of codes into categories, and then combining categories in broad themes corresponding to domains of life. All meaning units, categories, and domains received digital numbers. Note that the word “code” refers in this sense to specific verbal utterances forming meaning units (which then also received digital numbers used in quantitative analyses). The development of the coding system was done separately for the “what” (meaning sources) and “why” (motivations for meaning sources) questions following the same steps as indicated above. Interestingly, the same domains of life emerged for both the meaning sources and motivations for meaning sources. The broad domains of life covered in this coding system are similar to the domains and facets described in the work of the Whoqol Group. (2004) investigating the perceived quality of life. The domains included in the EHHI coding system are family, work, standard of living, interpersonal relationships (e.g., friends, close others but not family or community), health, personal life, leisure, spirituality/religion, society issues, community issues, and life in general. Because of the few references to the society and community domains of life and because participants might have had problems in distinguishing these two domains, the responses in these domains have been combined in our analyses and results.

Trained coders coded the responses to the questions on meaning sources and motivations for meaning sources according to the EHHI manualized coding system. Coders were instructed to assign one code to each of the responses on the meaning sources question (recall that participants were asked to list three meaning sources), which implies that each participant could have had a maximum of three codes for meaning sources (less if the participant mentioned fewer than three sources). For the motivations for meaning sources question, coders had to assign a maximum of two codes to each of the responses, which means that each participant could have had a maximum of six codes indicating motivations for meaning sources (recall the participants had to provide reasons why each of the three meaning sources they identified was meaningful to them). Because the data were available on code level after coding was done, we used lookup functions in Excel to add variables that represent the *what* and *why* categories and domains to which each code belongs. One trained coder coded the Ghanaian data, and another trained coder coded the South African data. Spot checks of coding were performed by the first two authors.

Using SAS 9.4, we first determined what percentage of sources of meaning was in each of the domains of life distinguished in the EHHI coding system which revealed the relative importance of the various relational domains/types of relationships. Although other life domains were also prominent in participants’ meaning sources (e.g., work), we focused for the rest of the analyses specifically on the reasons/motivations provided for relational meaning sources, that is, the motivations for meaning sources in the family, interpersonal relationships, spirituality/religion, and community/society domains. For each sample, we will report the five most frequent domains mentioned in motivations, the 10 most frequent motivation categories, and the 10 most frequent motivation codes for meaning sources in each of the four relational domains, respectively. Thus, only the most prominent domains, categories, and codes are shown. Note that, because the same domains emerged in the development of the formalized EHHI coding system for meaning sources and motivations for meaning sources, it is possible that, for example, “family” could be coded as both a source of meaning and a motivation for a source of meaning reflecting the intrinsic importance of the domain.

## Results

### The Frequency of Life Domains as Sources of Meaning

Table 2 presents the percentage of meaning sources found in the various life domains as captured in the EHHI coding system. The bottom row shows the total percentage of responses in the four relational domains (family, interpersonal relations, spirituality/religion, and community/society) combined.

**TABLE 2:** Percentages of life domains as meaning sources.

Domain	Ghana (%) (*f* = 1,073)	Multicultural SA (%) (*f* = 1,710)	Setswana SA (rural) (%) (*f* = 1,480)	Setswana SA (urban) (%) (*f* = 1,055)
Family	30.01	37.08	24.39	44.36
Work	21.06	14.50	7.77	3.89
Spirituality/religion	15.94	13.16	5.47	23.41
Health	8.11	5.56	2.23	0.66
Education	6.52	2.34	2.09	0.57
Standard of living	5.96	3.22	15.27	10.90
Personal life	4.57	7.08	9.80	2.94
Interpersonal relations	3.73	11.46	8.65	2.46
Life in general	1.86	1.87	21.28	7.68
Community/society	1.68	1.99	2.57	2.75
Leisure/free time	0.56	1.75	0.27	0.38
No meaning; death	0.00	0.00	0.20	0.00
Combined percentage of relational domains^a^	51.36	63.69	41.08	72.98

*SA, South Africa; f, total number of codes assigned to participants’ meaning sources for the sample. Domains are ordered from most frequent to least frequent according to the Ghanaian sample. ^a^In the last row, we added the percentages of the relational domains, that is, family, interpersonal relations, spirituality/religion, and community/society.*

From Table 2, it is clear that relationships were important sources of meaning for participants from all the groups, with family being the domain with the highest frequency in all samples. Spirituality/religion was also notably prominent as a source of meaning except for sample 3. Community and societal relationships were the least frequently mentioned of all relational domains (types of relationships).

### Most Frequent Domains, Categories, and Codes in the Motivations for Relationships as Sources of Meaning

Tables 3, 5, 7, 9 show the most frequent domains and categories, and Tables 4, 6, 8, 10 the most frequent codes that occurred in the motivations participants provided for relationships being important (Tables 3, 4 for the family domain, Tables 5, 6 for the interpersonal relations domain, Tables 7, 8 for the spirituality/religion domain, and Tables 9, 10 for the community/society domain). As described above, the verbatim responses of participants to the questions of *what* the most important sources of meaning are for them and also for *why* these sources are important, i.e., motivations or reasons for the meaning sources, were first coded, followed by the categorization of the codes, and thereafter clustered under life domains including various types of relational domains. Life domains and categories are thus abstracted groupings similar to themes and subthemes in narrative qualitative analyses, whereas the codes refer to the specific verbalizations as captured in the manualized EHHI codebook. In the following section, the findings will be reported by first referring to the abstracted domains and categories and then illustrated in verbatim quotes captured in codes.

**TABLE 3:** Percentages of the most frequent domains and categories mentioned in the motivations for meaning sources in the family domain of life.

Ghana (*f* = 272)		Multicultural SA (*f* = 757)		Setswana SA (rural) (*f* = 381)		Setswana SA (urban) (*f* = 514)	
Description	%	Description	%	Description	%	Description	%
**Domains**							
Family	58.82	Personal life	50.07	Family	68.24	Family	68.87
Personal life	31.25	Family	34.35	Personal life	26.25	Personal life	22.18
Interpersonal relations	5.51	Life in general	7.93	Interpersonal relations	2.10	Life in general	4.28
Standard of living	1.84	Interpersonal relations	3.96	Life in general	1.84	Spirituality/religion	1.75
Spirituality/religion	1.84	Spirituality/religion	2.77	Standard of living	0.79		
**Categories**							
Family−personal contribution	21.69	Personal life−support	17.04	Family−personal reward	24.15	Family−personal contribution	26.26
Family−intrinsic value/meaning	18.75	Personal life−value/meaning	16.12	Family−personal contribution	19.16	Family−personal reward	18.68
Personal life−support	16.91	Family−personal contribution	11.10	Personal life−support	15.49	Personal life−support	13.81
Family−sharing/reciprocity	9.56	Family−sharing/reciprocity	10.70	Family−WB of family members	13.39	Family−sharing/reciprocity	12.26
Personal life−joy/happiness/pleasure emotions	7.72	Life in general	7.93	Family−sharing/reciprocity	6.56	Family−WB of family members	6.42
Family−personal reward	7.35	Family−personal reward	5.81	Family−Intrinsic value/meaning	4.99	Family−Intrinsic value/meaning	5.25
Interpersonal relations−personal rewards	4.41	Personal life−joy/happiness/pleasure emotions	5.15	Personal life−value/meaning	4.99	Life in general	4.28
Personal life−value/meaning	3.31	Family−intrinsic value/meaning	4.49	Personal life−growth/engagement	2.36	Personal life−value/meaning	3.31
Standard of living	1.84	Family−WB of family members	2.25	Life in general	1.84	Personal life−joy/happiness/pleasure emotions	2.14
		Spirituality/religion−faith cultivation	2.25	Personal life−joy/happiness/pleasure emotions	1.05	Personal life−growth/engagement	1.75

*SA, South Africa. f, total number of codes assigned to participants’ motivations for meaning sources in the family domain of life. Where fewer than five domains or fewer than 10 categories are listed, addition of more domains/categories would have necessitated the presentation of more than five domains or more than 10 categories, because more domains/categories occurred in the dataset with the same frequency. We avoided the presentation of more than five domains or more than 10 categories because of space restrictions. Note that the percentages do not add up to 100, because only the most frequent domains/categories are presented.*

**TABLE 4:** Percentages of the most frequent codes in the motivations for meaning sources in the family domain of life.

Ghana (*f* = 272)		Multicultural SA (*f* = 757)		Setswana SA (rural) (*f* = 381)		Setswana SA (urban) (*f* = 514)	
Description	%	Description	%	Description	%	Description	%
To get support from it; support for personal growth; support in facing difficulties	16.54	To get support from it; support for personal growth; support in facing difficulties	13.74	To be looked after by children at old age	17.85	To get support from it; support for personal growth; support in facing difficulties	13.23
Awareness of having a beautiful/wonderful family	12.13	The most important thing/very important in own life	7.00	To get support from it; support for personal growth; support in facing difficulties	16.01	To be looked after by children at old age	9.34
Source of joy, positive/wonderful feelings; cheerfulness	7.35	To give meaning to life; without it nothing has meaning; to make life worth living	6.34	Love for children	5.51	Love for children	9.14
Love for children	5.51	To get refuge, security, comfort; a safe harbor	3.30	To promote own children’s development/growth	4.46	To enjoy the presence of children	3.89
To give support to the family/altruism toward the family	5.15	Source of joy, positive/wonderful feelings; cheerfulness	2.64	The most important thing/very important in own life	3.94	Sibling relationships	3.50
To have a family	3.68	To get love from family	2.38	Love/respects for parents/family of origin	2.89	To have received unconditional love from parents/teachings from them/life	3.11
Feeling of belonging/integration	3.68	Feeling/emotion of happiness	2.38	Good education for children	2.62	Sharing with nephew/niece; special bonding	3.11
Responsibility/care as a parent; not to become a burden for children	2.94	It is everything	2.38	Good job for children	2.36	To promote own children’s development/growth	2.72
Family happiness; to share happiness	2.57	Love sharing within family	2.11	It is necessary for the family	1.84	The most important thing/very important in own life	2.72
				To enjoy the presence of children	1.84	Love/respects for parents/family of origin	2.53

*SA, South Africa; f, total number of codesassigned to participants’ motivations for meaning sources in thefamily domain of life. Where fewer than 10 codes are listed, additionof more codes would have necessitated the presentation of more than 10 codes, because more codes occurred in the dataset with the same frequency. We avoided the presentation of more than 10 codes due to space restrictions. Note that the percentages do not add up to 100, because only the most frequent codes are presented.*

**TABLE 5:** Percentages of the most frequent domains and categories in the motivations for meaning sources in the interpersonal relations domain of life.

Ghana (*f* = 32)		Multicultural SA (*f* = 265)		Setswana SA (rural) (*f* = 120)		Setswana SA (urban) (*f* = 29)	
Description	%	Description	%	Description	%	Description	%
**Domains**							
Interpersonal relations	53.13	Interpersonal relations	49.81	Interpersonal relations	75.00	Interpersonal relations	68.97
Personal life	31.25	Personal life	37.74	Personal life	11.67	Personal life	13.79
Family	9.38	Life in general	4.15	Family	4.17	Community/society issues	6.90
Community/society issues	6.25	Family	3.02	Community/society issues	4.17	Life in general	6.90
		Spirituality/religion	1.89	Life in general	3.33	Health(physical)	3.45
**Categories**							
Interpersonal relations−personal rewards	21.88	Interpersonal relations−sharing/reciprocity	16.98	Interpersonal relations−harmony, balance	27.50	Interpersonal relations−personal rewards	24.14
Interpersonal relations−intrinsic value/meaning	15.63	Personal life−support	15.47	Interpersonal relations−personal rewards	17.50	Interpersonal relations−sharing/reciprocity	20.69
Interpersonal relations sharing/reciprocity	15.63	Interpersonal relations−Personal rewards	14.34	Interpersonal relations−sharing/reciprocity	15.83	Interpersonal relations−personal contribution	20.69
Personal life−growth/engagement	9.38	Interpersonal relations−Intrinsic value/meaning	11.70	Interpersonal relations—intrinsic value/meaning	10.83	Life in general	6.90
Personal life—joy/happiness/pleasure emotions	9.38	Interpersonal relations—Personal contribution	6.42	Personal life—value/meaning	5.00	Personal life—support	6.90
Family—intrinsic value/meaning	6.25	Personal life—joy/happiness/pleasure emotions	5.66	Life in general	3.33		
Community/society issues—personal contribution	6.25	Personal life—value/meaning	4.91	Interpersonal relations—personal contribution	3.33		
		Life in general	4.15				
		Personal life—fullness/awareness	2.26				

*SA, South Africa; f, total number of codesassigned to participants’ motivations for meaning sources in theinterpersonal relations domain of life. Where fewer than five domainsor fewer than 10 categories are listed, addition of moredomains/categories would have necessitated the presentation of morethan five domains or more than 10 categories, because more domains/categories occurred in the dataset with the same frequency. We avoided the presentation of more than five domains or more than 10 categories due to space restrictions. Note that the percentages do not add up to 100, because only the most frequent domains/categories are presented.*

**TABLE 6:** Percentages of the most frequent codes in the motivations for meaning sources in the interpersonal relations domain of life.

Ghana (*f* = 32)		Multicultural SA (*f* = 265)		Setswana SA (rural) (*f* = 120)		Setswana SA (urban) (*f* = 29)	
Description	%	Description	%	Description	%	Description	%
To get help, support from friends/neighbors	12.50	To get support from it; support for personal growth; support in facing difficulties	10.90	Harmony, balance in interpersonal relations	27.50	To love others	13.79
Source of joy, positive/wonderful feelings; cheerfulness	9.38	To get help, support from friends/neighbors	4.14	To get understanding from friends	10.83	To get help, support from friends/neighbors	13.79
Importance of friendship	6.25	To share experiences/good and bad moments	3.76	To respect each other	9.17	To enjoy being together	6.90
Better interpersonal relationships	6.25	Importance of friendship	3.38	Trust in friends/others	5.00	To help each other	6.90
To share experiences/good and bad moments	6.25	Source of joy, positive/wonderful feelings; cheerfulness	3.01	To get help, support from friends/neighbors	4.17	To respect each other	6.90
Personal contribution to the community	6.25	To enjoy being together	2.63	To help each other	3.33	To get understanding from friends	6.90
		To help each other	2.63	To love others	3.33	To get support from it; support for personal growth; support in facing difficulties	6.90
		Feeling of belonging/integration	2.63	Better interpersonal relationships	2.50		
		To get refuge, security, comfort; a safe harbor	2.63	To reciprocally give and take	2.50		
				The most important thing/very important in own life	2.50		

*SA, South Africa; f, total number of codes assigned to participants’ motivations for meaning sources in the interpersonal relations domain of life. Where fewer than 10 codes are listed, addition of more codes would have necessitated the presentation of more than 10 codes, because more codes occurred in the dataset with the same frequency. We avoided the presentation of more than 10 codes due to space restrictions. Note that the percentages do not add up to 100, because only the most frequent codes are presented.*

**TABLE 7:** Percentages of the most frequent domains and categories in the motivations for meaning sources in the spirituality/religion domain of life.

Ghana (*f* = 148)		Multicultural SA (*f* = 287)		Setswana SA (rural) (*f* = 75)		Setswana SA (urban) (*f* = 273)	
Description	%	Description	%	Description	%	Description	%
**Domains**							
Spirituality/religion	74.32	Personal life	48.08	Spirituality/religion	65.33	Spirituality/religion	56.04
Personal life	21.62	Spirituality/religion	40.42	Personal life	25.33	Personal life	33.7
Standard of living	1.35	Life in general	5.92	Life in general	5.33	Life in general	7.33
		Family	1.74	Family	2.67	Family	1.1
				Work	1.33	Work	0.73
**Categories**							
Spirituality/religion—faith cultivation	62.84	Spirituality/religion—faith cultivation	36.59	Spirituality/religion—faith cultivation	53.33	Spirituality/religion—faith cultivation	49.08
Spirituality/religion—spiritual growth	10.14	Personal life—value/meaning	21.95	Personal life_ support	12.00	Personal life—support	12.45
Personal life—value/meaning	7.43	Life in general	5.92	Personal life—value/meaning	8.00	Life in general	7.33
Personal life—support	2.70	Personal life—harmony/balance	5.92	Spirituality/religion—religious practice/witness	8.00	Personal life—competence/mastery	7.33
Personal life—purpose	2.70	Personal life—support	4.88	Life in general	5.33	Spirituality/religion—spiritual growth	4.4
Personal life—competence/mastery	2.70	Personal life—optimism	3.14	Spirituality/religion—spiritual growth	4.00	Personal life—value/meaning	2.93
Personal life—joy/happiness/pleasure emotions	2.03	Personal life—competence/mastery	2.79	Personal life—competence/mastery	2.67	Spirituality/religion—religious practice/witness	2.56
Personal life—no negative feelings	2.03	Spirituality/religion—spiritual growth	2.79			Personal life—growth/engagement	2.2
Standard of living	1.35	Personal life—growth/engagement	2.09			Personal life—positive experiences/inner states of well-being	2.2
Spirituality/religion_ religious practice/witness	1.35	Personal life—fullness/awareness	2.09			Personal life—joy/happiness/pleasure emotions	1.83

*SA, South Africa; f, total number of codes assigned to participants’ motivations for meaning sources in the spirituality/religion domain of life. Where fewer than five domains or fewer than 10 categories are listed, addition of more domains/categories would have necessitated the presentation of more than five domains or more than 10 categories, because more domains/categories occurred in the dataset with the same frequency. We avoided the presentation of more than five domains or more than 10 categories due to space restrictions. Note that the percentages do not add up to 100, because only the most frequent domains/categories are presented.*

**TABLE 8:** Percentages of the most frequent codes in the motivations for meaning sources in the spirituality/religion domain of life.

Ghana (*f* = 148)		Multicultural SA (*f* = 287)		Setswana SA (rural) (*f* = 75)		Setswana SA (urban) (*f* = 273)	
Description	%	Description	%	Description	%	Description	%
Mercy we receive from God; to receive God’s blessing	13.51	To give meaning to life; without it nothing has meaning; to make life worth living	8.36	To live up to my purpose as a believer; to serve/witness/please/honor God	17.33	We get everything from God; it’s God’s gift	17.22
We get everything from God; it’s God’s gift	12.16	“Point of reference” in life; without it everybody is lost	8.36	To get inspiration for living daily life	10.67	To live up to my purpose as a believer; to serve/witness/please/honor God	12.45
Importance of getting close to God	11.49	To live up to my purpose as a believer; to serve/witness/please/honor God	5.23	Importance of getting close to God	8.00	To get support from it; support for personal growth; support in facing difficulties	9.16
For my eternal life	6.76	For my eternal life	5.23	The most important thing/very important in own life	6.67	Source of interior strength/power	6.96
Spiritual growth	6.76	We get everything from God; it’s God’s gift	5.23	To get support from it; support for personal growth; support in facing difficulties	6.67	Trust in the Lord	5.49
Love for God	6.08	Importance of getting close to God	4.88	We get everything from God; it’s God’s gift	6.67	To get inspiration for living daily life	3.66
Basis of individual life	5.41	Inner peace	4.53	Better knowledge of my religion	6.67	Necessary to solve all problems	3.66
To realize God’s purpose/project on me	3.38	To get inspiration for living daily life	4.53	To get refuge, security, comfort; a safe harbor	5.33	To get refuge, security, comfort; a safe harbor	3.30
God; God exists	3.38			Trust in the Lord	4.00	Importance of getting close to God	3.30

*SA, South Africa; f, total number of codes assigned to participants’ motivations for meaning sources in the spirituality/religion domain of life. Where fewer than 10 codes are listed, addition of more codes would have necessitated the presentation of more than 10 codes, because more codes occurred in the dataset with the same frequency. We avoided the presentation of more than 10 codes due to space restrictions. Note that the percentages do not add up to 100, because only the most frequent codes are presented.*

**TABLE 9:** Percentages of the most frequent domains and categories in the motivations for meaning sources in the community/society domain of life.

Ghana (*f* = 17)		Multicultural SA (*f* = 45)		Setswana SA (rural) (*f* = 35)		Setswana SA (urban) (*f* = 32)	
Description	%	Description	%	Description	%	Description	%
**Domains**							
Community/society issues	41.18	Community/society issues	37.78	Interpersonal relations	42.86	Community/society issues	68.75
Personal life	23.53	Personal life	28.89	Community/society issues	37.14	Personal life	18.75
Spirituality/religion	17.65	Family	11.11	Personal life	14.29	Interpersonal relations	9.38
Work	11.76	Interpersonal relations	8.89	Leisure/free time	2.86	Spirituality/religion	3.13
Interpersonal relations	5.88	Life in general	6.67	Life ingeneral	2.86		
**Categories**							
Community/society issues—personal contribution	29.41	Community/society issues—personal contribution	26.67	Interpersonal relations—personal rewards	22.86	Community/society issues—personal contribution	65.63
Spirituality/religion—faith cultivation	17.65	Family—personal contribution	8.89	Community/society issues—personal contribution	20.00	Personal life—support	15.63
Personal life— satisfaction/achievement/gratification	11.76	Life in general	6.67	Community/society issues—welfare	14.29	Interpersonal relations—personal rewards	9.38
Community/society issues—welfare	11.76	Personal life—joy/happiness/pleasure emotions	6.67	Interpersonal relations—harmony/balance	11.43	Personal life—growth/engagement	3.13
Work- intrinsic value/meaning	5.88	Interpersonal relations—sharing/reciprocity	4.44	Interpersonal Relations_ sharing/reciprocity	8.57	Spirituality/religion—faith cultivation	3.13
Work-satisfaction/achievement	5.88	Personal life—growth/engagement	4.44	Personal life_ value/meaning	5.71	Community/society issues—personal reward/integration	3.13
Interpersonal relations—intrinsic value/meaning	5.88	Personal life—value/meaning	4.44				
Personal life—joy/happiness/pleasure emotions	5.88	Community/society issues—welfare	4.44				
Personal life—support	5.88	Community/society issues—personal reward/integration	4.44				

*SA, South Africa; f, total number of codes assigned to participants’ motivations for meaning sources in the community/society domain of life. Where fewer than five domains or fewer than 10 categories are listed, addition of more domains/categories would have necessitated the presentation of more than five domains or more than 10 categories, because more domains/categories occurred in the dataset with the same frequency. We avoided the presentation of more than five domains or more than 10 categories due to space restrictions. Note that the percentages do not add up to 100, because only the most frequent domains/categories are presented.*

**TABLE 10:** Percentages of the most frequent codes in the motivations for meaning sources in the community/society domain of life.

Ghana (*f* = 17)		Multicultural SA (*f* = 45)		Setswana SA (rural) (*f* = 35)		Setswana SA (urban) (*f* = 32)	
Description	%	Description	%	Description	%	Description	%
To live up to my purpose as a believer; to serve/witness/please/honor God	17.65	To help other people; people in need	15.56	To get help, support from friends/neighbors	20.00	Love for the neighbor; brotherhood	62.50
To help other people; people in need	17.65	Personal contribution to the community	11.11	Harmony, balance in interpersonal relations	11.43	To get support from it; support for personal growth; support in facing difficulties	15.63
To get fulfillment	11.76	To promote own children’s development/growth	4.44	Love for the neighbor; brotherhood	11.43	To get help, support from friends/neighbors	6.25
Personal contribution to the community	11.76	To enrich personal life	4.44	To help each other	8.57	To be aware of people’s wickedness	3.13
		Source of joy, positive/wonderful feelings; cheerfulness	4.44	Personal contribution to the community	5.71	To get refuge, security, comfort; a safe harbor	3.13
				Peace in the society/community	5.71	To live up to my purpose as a believer; to serve/witness/please/honor God	3.13
						To help other people; people in need	3.13
						To feel integrated/a part of society	3.13

*SA, South Africa; f, total number of codes assigned to participants’ motivations for meaning sources in the community/society domain of life. Where fewer than 10 codes are listed, addition of more codes would have necessitated the presentation of more than 10 codes, because more codes occurred in the dataset with the same frequency. We avoided the presentation of more than 10 codes due to space restrictions. Note that the percentages do not add up to 100, because only the most frequent codes are presented.*

#### Most Frequent Domains, Categories, and Codes in the Motivations for Family as Source of Meaning

See Tables 3, 4 for the results of the most frequent domains, categories, and codes as found for motivations in the Family domain as important source of meaning.

The vast majority of motivations for family being an important source of meaning emerged from references to facets associated with the family and personal life domains as mentioned in answers to the *why* question. Notably, family was the domain most frequently referred to in motivations for all groups, except for the multicultural South African group where personal life was the domain containing the most motivations. Important motivation categories that emerged across the samples were that participants felt that they could contribute to their family by, for example, loving them, caring for them, respecting them, and supporting them; that they experienced support from these relationships; that they get some form of personal reward from family members, for example, by getting love from family members, being looked after by children at old age, or enjoying the presence of children; that family gave them a sense of sharing and reciprocating, for example, love and happiness; and that family is intrinsically meaningful or provides meaning and value to their lives. Other top life domains mentioned in the motivations for the importance of family as a source of meaning were spirituality, standard of living, interpersonal relations, and life in general.

On code level, prominent motivations for the family as a source of meaning included that participants experienced that they get support (including support to grow personally and support when facing difficulties) from their family members and that they love their children. For the two older Setswana groups, being looked after by children at old age was a frequently occurring code, whereas experiencing family as a source of joy and positive feelings was a common code among the Ghanaian and multicultural South African groups.

#### Most Frequent Domains, Categories, and Codes in the Motivations for Interpersonal Relations as Source of Meaning

Table 5 presents the most frequent domains and categories abstracted from the motivations provided for the interpersonal relations domain as source of meaning, whereas Table 6 reflects the specific motivation codes.

For all groups, the domain that featured most in participants’ motivations for (non-family) interpersonal relations being important was the interpersonal relations domain itself. Personal life was the second most common motivation domain. On category level, motivation categories that emerged frequently across the groups included getting personal rewards in the form of, for example, getting help, support, or understanding from friends or neighbors or having a sense of belongingness or integration; experiencing support; sharing among others experiences or good and bad times, to enjoy being together, or to help or respect each other; experiencing harmony or balance in interpersonal relations; and contributing to others’ lives by, for example, loving them. Common codes across all groups included getting help or support from friends or neighbors; getting support (including support to grow personally and support when facing difficulties) from others, helping each other; and enjoying being together or experiencing the relationships as being a source of joy and positive feelings. Notably, for the rural Setswana-speaking groups, experiencing harmony and balance in interpersonal relations was an important motivation.

#### Most Frequent Domains, Categories, and Codes in the Motivations for Spirituality/Religion as Source of Meaning

See Tables 7, 8 for the most frequent domains, categories, and codes for the motivations of spirituality/religion being a source of meaning, as mentioned in answers to the *why* question.

In the reasons for spirituality/religion being an important source of meaning to people, spirituality/religion and personal life were the most frequently occurring domains mentioned in the reasons for importance, with spirituality/religion being the most frequent domain for all groups but the multicultural South African sample. Across all groups, faith cultivation was by far the most frequent category emerging in participants’ motivations for spirituality/religion being meaningful. This category included responses such as living up to one’s purpose by serving, witnessing, pleasing, or honoring God, receiving God’s mercy or blessing, getting everything from God or experiencing the specified source of meaning as God’s gift, emphasizing the importance of getting close to God, eternal life, loving God, realizing God’s purpose, getting inspiration for living daily life, or trusting in the Lord. Other prominent motivation categories across the groups were that spirituality/religion provided participants with value or meaning in their lives and that it provided them with personal identity and a sense of harmony. Prominent codes included expressing that we get everything from God or that life is God’s gift; living up to one’s purpose by serving, witnessing, pleasing, or honoring God; the importance of getting close to God; and to get inspiration for living daily life.

#### Most Frequent Domains, Categories, and Codes in the Motivations for Community/Society as Source of Meaning

Tables 9, 10 reflect the most frequent domains, categories, and codes captured for the motivations for the importance of community/society as source of meaning.

First, it is important to note that the total number of motivations for community/society being a source of meaning was small for all samples, especially for the Ghanaian sample where only 17 codes were assigned to motivations for community/society as a source of meaning. Interpretation should therefore be done with caution. The most common domains in which motivations fell, in this case, were community/society itself and personal life. Interestingly, for the rural Setswana-speaking sample, interpersonal relations were the most frequent domain. On category level, a category that emerged prominently across all samples was personally contributing to the community by, for example, helping or loving others. Codes that commonly occurred across the samples included helping others or helping people in need and personally contributing to the community.

## Discussion

The present study aimed to determine the relative importance of various types of relationships as important sources of meaning for people in African samples and to explore the motives for meaning in relational domains as manifested in four samples from Ghana and South Africa. The motives for meaning were explored in the relational domains of family, interpersonal relations (close but not family), spirituality/religion, and community/society. The findings of the study showed that relational domains of life made up a large proportion of sources of meaning for participants in all samples, with community/society occurring the least. Most important relational types showed not only some similarities with findings in the West, but also differences. Motives for sources of meaning in the relational domains revealed, apart from domain-informed relational reasons, also personal experiences and needs, reflecting a dynamic dialectical pattern of individual and collectivist experiences. Findings suggest a reconsideration of traditional assumptions about collectivism in the African context. Altogether, the insight into philosophical assumptions in the African context provided by findings regarding sources of meaning and the motivations for their importance offers a scientific basis for appropriate well-being interventions in African contexts. These findings will be discussed in more depth below in the light of relevant literature.

### Life Domains as Sources of Meaning

The four relational domains (family, interpersonal relations, spirituality/religion, and community/society) together made up a large proportion of meaning sources across the 11 domains of life included in the coding system, namely, 51.36%, 63.69%, 41.08%, and 72.98%, respectively, in the cases of samples 1, 2, 3, and 4. The fact that the rural Setswana group showed a relatively lower percentage of meaning sources in the four relational domains may be because some relational meaning sources might have been allocated to the life-in-general domain, which is much higher in this sample than in the other samples. This finding reflects an integrated, more holistic experience of meaning by this rural, more indigenous/traditional sample of participants. Among this group, this finding also reflects the notion that relationships might be seen as encompassing all aspects of an individual’s life.

Our finding that family is the most often mentioned relational context providing meaning is in line with previous research (e.g., Delle Fave et al., 2011). Note that notions of what is meant by “family” may differ in various contexts (e.g., extended family vs. core family or as “all putting their hands into the same pot to eat” as is often the case in an African context). The importance of family has been echoed in similar studies in the African context (Mason, 2013; Wissing et al., 2014), where the family was mostly associated with the support that is expected to be received.

The spiritual/religion domain of life is the second most prominent relational domain to provide meaning to life in the case of three samples as could be expected considering the importance attached to spirituality in African philosophical perspectives (cf., Onyedinma and Kanayo, 2013; Nwoye, 2017; Ohajunwa and Mji, 2018). This is different from findings in other, mostly Western samples as reported by Delle Fave et al. (2011), where spirituality/religion is less prominent. Surprisingly, spirituality/religion was, in contrast to the other (urban) African samples, not so prominently mentioned in the case of the rural Setswana sample. In the latter instance, participants might have integrated spiritual aspects in a more holistic contextual life-in-general experience as indicated above, a life domain that occurred frequently for this group. The minimal reference to spirituality among the rural sample might also reveal the taken-for-granted assumption that experiencing meaning is synonymous to being spiritual or religious because of the pervasive nature of spirituality in African indigenous world views. However, as the two PURE groups also had the lowest percentage of participants indicating that they belong to a religious group, further in-depth qualitative interviews are necessary to clarify this finding.

The lowest percentage of meaning sources in the relational domains was ascribed to the community/society domain of life. This is also surprising as it could have been expected that communities are of particular importance in collectivist African groups. It is, however, possible that facets of community/society are accepted as so self-evident or as an integral part of all other aspects of life and that it was not mentioned as standing out. Nevertheless, the finding that community/society is seldom mentioned as a source of meaning in life is in line with the findings of a multicountry study by Delle Fave et al. (2011). This may reflect a global stronger inner-circle solidarity. Another possible explanation is that in contexts such as Ghana, the lines between family and/or close relations and the community are blurred as kinship ties are formed without any blood connections (Van der Geest, 1998; Wilson et al., 2017), and meaning in the wider community/society is thus also included in a broad conception of family. It is also likely that when individuals reflect on their relationships, greater reference is made to tangible connections including family, friends, and acquaintances, rather than the broader community. In a sense, my community is my friends, neighbors, and family. It may also be that the larger community and society carry more political connotations and are therefore considered to be farther away. The reasons for the relatively low importance of community as life domain providing meaning as found in this study in an African context need to be further explored and explained given existing African value systems and possible shifts in perspectives. Nonetheless, our findings indicate that notions of collectivism in African contexts cannot be generalized to automatically include communities and society at large.

Our findings about the huge role played by relational domains in all four African samples can also be contextualized further by noting that work was the second most important life domain providing meaning in the case of the more educated and younger Ghanaian and South African samples, whereas standard of living was the third most prominent life domain reflected in sources of meaning in the case of the older, less educated Setswana samples. The latter groups with their lower levels of education probably struggle more with material needs, which can explain why several participants provided reasons for relationships being important such as to be looked after by children in old age, but also wish to have something to give away to others (response not shown in above tables that include only the highest rankings). Although work and standard of living are not relational life domains, it sometimes happens that relational motives are provided when work or standard of living is listed as sources of meaning. For example, where work is mentioned in people’s sources of meaning, the reasons for the importance of work sometimes refer to relationships with work colleagues, or where the standard of living is a source of meaning, the motive is sometimes that it enables the participant to care for the family as found by Wissing et al. (2019a) in a multicultural South African group. Dzokoto et al. (2019) also indicated that work-related meaning was linked to personal growth and the greater good, which has some underlying relational implications.

### Motivations for Meaning Sources in Relational Domains

Our findings across all four relational domains (family, interpersonal relations [close, but not family, e.g., friends, neighbors], spirituality/religion, and community/society) indicated that the motivations for sources of meaning are described primarily in domain-specific qualities as can be expected, but also in terms of intrapersonal descriptors as classified under the personal life domain. Across relational domains, domain-typical reasons referred to, for example, contributions made to or rewards received from that relationship, sharing/reciprocity in the relationship, support needed or given, the intrinsic value of that bond, nurturing that valued bond, and harmony experienced in the context of that domain of life. These reasons resonate strongly with African perspectives on the importance and quality of interpersonal relationships.

Noteworthy, our findings showed that many motivations for the things that are meaningful to people emerged from a personal experience or perspective as classified in our scoring system under the personal life domain. Such personal motives include resonance experienced with own value system, support felt, personal growth experienced, the experience of satisfaction, happiness, joy, pleasure, inner state of well-being, or a sense of competence and mastery. The fact that personal life plays such an important role in the motivations for meaning reflects an internal, self-reflective stance not commonly described in traditional African perspectives. It can, of course, also be argued that many categories and codes included in the personal life domain represent facets and responses (as captured in the EHHI coding system) that implicitly reflect relationships to others, such as the experience of support, harmony, and loving feelings.

The noted importance of personal experiences as motives for meaning sources may point in the direction of a greater individualism than what was thought before. It is different from assumptions in the past for the African context. This finding may be in line with a more global phenomenon of cultural shifts as described in the Inglehart–Welzel model and shown on the world cultural map^1^. Inglehart and Welzel showed shifts in world cultural patterns as manifested in pattern changes on the dimensions of survival versus self-expression values, and traditional versus more secular–rational values. The second marker on both dimensions is linked to individualism and a more independent relational orientation. Longitudinal evidence from the World Values Studies (WVS) based on Schwartz’s model showed that emancipative orientations including mostly self-expression values are rising across all countries (Schwartz, 2006; Welzel, 2010). Several such self-expression verbalizations are part of the EHHI coding system included in the personal life domain and found in the present study, for example, referring to growth, mastery, autonomy, self-actualization, and values.

Although there is a general agreement that self-expression values associated with individualism are rising across the world, there are different interpretations of what this increase means (Welzel, 2010). On the one hand, self-expression and individualism are seen as uncivic and an indication of egoism and selfishness. But on the other hand, the rising self-expression orientation is interpreted as a civic orientation and linked to altruism and strong social capital. Welzel’s (2010) analysis of empirical data affirms the latter interpretation. He found that self-expression values (as described in the WVS) were linked to altruism, in particular in the case of high levels of these values, and that self-expression values as evaluated in the WVS are linked to peaceful collective action. He concluded that self-expression values are “a civic form of modern individualism” (Welzel, 2010, p. 152). Following this lead, we interpret our present findings as reflecting an African individualism, which is also linked to an appreciation of a greater good. But, as people and societies change, the developments referred to by Inglehart and Welzel may also change again as suggested by Inglehart (2018), who refers to the reverse of these processes by an upcoming authoritarian populist trend.

The trend we noticed toward seemingly more individualistic intrapersonal motives in meaning ascriptions in relational domains is still embedded in contextual relatedness and reflects meaning detection or meaning construction in widening circles from the inside outward through coordination with others and context. The intrapersonal motives for meaning are accompanied by external expectations of reciprocity and accountability to “live well” to “have the capacity to do good.” In this sense, the construction of meaning is not only an individual process but also arises through the understanding of one’s place in the world and the grand scheme of things. This argument is important because it goes beyond the existing dichotomy of individualism and collectivism to present an integrated approach that views the individual not only as a person but also as a person “in a context.” This perspective dovetails with that of Dzokoto et al. (2019). We hypothesize that our findings reflect a (perhaps modern) civic form of individualism that coexists with characteristics of collectivism (context relevantly expressed). We contend that individuals simultaneously hold individualist and collectivist notions in a “dialogical self” as described by Hermans (2001). This dialogue between individualist and collectivist motives within the self is then expressed in a dialectic, dynamic pattern that may change flowingly in various contexts and circumstances. In line with the work by Singelis (1994), individuals’ images of self at the same time reflect an emphasis on connectedness and relations (interdependent self-construal) and separateness and uniqueness (independent self-construal).

### Theoretical Perspectives

Findings in this study indicate the importance of relationships as sources of meaning across domains of life. The quality of the motivations for what is important to people in these domains reflects the African philosophical notions on the value of relationships (Gyekye, 1996; Baloyi and Mokobe-Rabothata, 2014; Nwoye, 2015). The findings provide empirical evidence of the African notion of “Ubuntu” as well as Metz’s (2011, 2013) moral theory of human rights and dignity.

The interwovenness of individual and communal motives found in the present study resonates with Nyamnjoh’s (2015) idea of African conviviality and acceptance of incompleteness. Furthermore, our interpretation that a (perhaps modern) form of African individualism and collectivism exists in which features can be mixed and coexisting resonates with Singelis’s (1994) notion that interdependent and independent self-construals can and do coexist. It also dovetails with African philosophical ideas that seemingly opposites can coexist or become each other, or transform according to the requirements of the contexts or necessity (cf. Nyamnjoh, 2015). Nyamnjoh (2015) contends: “Just as there is more and less to bodies than meets the eye, and more and less to the eye than meets bodies, there is much more and much less to what strikes us in things or facets of things” (p. 5). This assumption of the “ever-shifting complexities of being and becoming” (Nyamnjoh, 2015, p. 5) is different from the typical Western dualistic modes of thinking about reality and transcendental aspects.

Findings can also be interpreted as showing that individual and communal–cultural experiences are integrated in a dialogical self as described in Hermans’ (2001) theoretical model in which he hypothesized that the self and culture can be seen as various positions among which there can be dialogical relationships. This dialogical relationship can be seen as expressed in a dynamic pattern as described by Adams and Markus (2001, 2004)− in this case reflecting dynamically integrated flowing notions of both individualism and collectivism. It is not clear whether this is a new phenomenon in these African contexts, or whether it already existed in the past but had just not been noticed and empirically illustrated as in the present study.

Another relevant theoretical model explaining the integration of meaning across levels of relatedness is the M&RW model as described by Wissing et al. (2019b). The M&RW model assumes a systems perspective of the world and humans and a robust relational ontology position (thus assuming the interconnectedness of all things). This model is visually depicted as a double spiral string or helix widening upward as in a cone, to seamlessly include broader and increasing complex relational systems (from intrapersonal, to interpersonal, social, and eventually spiritual/transcendental) providing meaning in, to, and of life. In this model, it is hypothesized that meanings are made in and among relationships/interconnectedness on intrapersonal, interpersonal, social, and spiritual levels while being anchored in a specific place and time. The present empirical findings confirm that relationships are important sources of meaning as experienced on personal, interpersonal (family and other close relationships), community/society, and spiritual levels. The interwovenness of individual and communion motives in relations as sources of meaning, as found in the present study, is an empirical manifestation of the seamless flow of information in the dynamic interactions among levels of reality as conceptualized in Wilson et al.’s (2019b) model.

The “ever-shifting complexities of being and becoming” as accepted in African worldviews (Nyamnjoh, 2015, p. 5) are captured in our interpretation of dialectic patterns of individualist and collectivist values. These values emerged empirically in the motives for the importance of various types of relationships. Such complexities and dialectic patterns need to be taken into account when interventions for the promotion of quality of life and well-being in these African contexts are considered. The current state-of-the-art individualistic focused strategies used in Western contexts may not be optimal in these African contexts, and neither an only communal-focused approach.

### Limitations

A limitation of this study is that the coders for the Ghanaian and South African samples were not the same people and that interrater reliabilities were not determined. This may limit the comparability of the findings between the two countries. This limitation is ameliorated to an extent by the use of a formalized codebook or manual by trained coders in both countries and by spot checks of coding by the first two authors. A further limitation is that the findings of this study are based on responses to short open-ended questions rather than in-depth interviews. The impact of this limitation is reduced by the large sample size. Richer qualitative data that are gathered by means of, for example, in-depth interviews may shed further light on the findings and conclusions and should be considered for future research. The inclusion of more variables for the description of samples (such as socioeconomic levels) might have facilitated explanation of some findings. Nevertheless, this study contributed to a deeper understanding of motives for meaning in relational domains as manifested in the traditionally collectivist African context pointing to a dynamic, dialectical pattern of individualistic and collectivistic values as manifested in the experiences of contemporary laypeople.

## Conclusion and Contributions

Relational domains of life are major sources of meaning to laypeople in two African countries, Ghana and South Africa, reflecting African philosophical notions of relational embeddedness. The relative importance of relational types/domains is in line with some findings in the West (importance of family), but also differed (spirituality more important in our African samples). Different from what might have been expected in a collectivist African context, communal/societal bonds were less important than close personal relationships. The motivations for the importance of these relational domains reflect, apart from strong relational domain–nuanced reasons, also a personal orientation enriching our understanding of contemporary laypeople’s experiences showing a dynamic pattern of integration of individual and collectivist values. In the African context, relational embeddedness blurs the lines between existing categorizations of different forms of relationships. Findings may, however, be in line with the globally noted cultural shifts taking place as indicated in the Inglehart–Welzel model and illustrated in the world cultural map. See also Welzel (2010), Inglehart (2018) showing a new kind of modern coexisting or mixed individualism–collectivism associated with a shift to stronger self-expression values. It is, however, not known if the dynamic pattern of individualist and collectivist notions noted in this study is a new recent phenomenon or just a phenomenon that already existed but was only now empirically noticed and described for the first time. Empirical findings from this study are aligned with Singelis’s view that interdependent and dependent self-construals can and do coexist; notions of a dialogical self as proposed by Hermans (2001); and propositions of the M&RW model (Wissing et al., 2019b), hypothesizing an interplay among intrapersonal, interpersonal, social, and transpersonal relationships in meaning-making while being anchored in a specific space and time.

This study contributes to a deeper understanding of motives for relational meaning sources as manifested in two African samples, highlighting a dynamic pattern of integrated individualistic and collectivist notions in meaning attributions and motives. Findings link to notions of a dialogical self not previously described in the African context and provide empirical support for facets of a recent African model of meaning and relatedness. Further research is necessary to understand the relatively low percentage of motives for meaning manifested in the community/society relational domain of life in all four samples. This percentage could have been expected to be higher given the traditionally described more collective cultural orientation in African countries. In-depth qualitative research is necessary to explore the tapestry of experiences that rural people had in mind when they referred to life in general and whether these experiences also included spiritual facets as suspected because of their relatively lower ranking of the spiritual/religious domain in comparison to the other samples. It can also be revisited to what extent the traditional (and sometimes stereotyping) notions of individualism and collectivism are still viable and applicable cultural explanatory concepts in the changing global landscape.

## Data Availability Statement

The datasets generated for this study are available on reasonable request to the corresponding author.

## Ethics Statement

The Health Research Ethics Committee of the North-West University, South Africa, approved the studies reported in samples 2, 3, and 4 (Approval Numbers: NWU00002-07-A2 and NWU-00016-10-A1), and the University of Ghana Ethics Committee for Human Research approved the study reported in sample 1 (Approval Number: ECH 086 16-17). The participants provided their written informed consent to participate in this study.

## Author Contributions

MW, AW, and LS conceptualized the study and supervised the coding and capturing of qualitative data. MW and LS collected the SA data as part of the funded FORT3 project led by MW. AW supervised the Ghanaian data collection. WS and LS conducted the statistical analyses. MW drafted the first version of the manuscript with major inputs into the literature review by AW and SC. LS and WS drafted the “Results” section and parts of the “Materials and Methods.” MW integrated author comments. LS supervised technical editing of the manuscript. All authors made inputs on the manuscript as a whole in following versions and approved the final version.

## Conflict of Interest

The authors declare that the research was conducted in the absence of any commercial or financial relationships that could be construed as a potential conflict of interest.

## Footnotes

1 https://en.wikipedia.org/wiki/Inglehart%E2%80%93Welzel_cultural_map_of_the_world
